# Prof. Bell, Climate of Texas for Surgical Work, Etc.

**Published:** 1894-09

**Authors:** 


### Medical News and Miscellany.

---

**Errata.**—In Dr. Oates' excellent article on "Malarial Hæmatinuria" in our July number, for "mercuric chloride" read "mercurus chloride." The error occurs in three places.

---

**Prof. Jas. H. Bell,** so long and favorably known as a teacher in Jefferson Medical College, and as an authority on diseases of the eye, which branch of study has been for years his specialty, has removed from Philadelphia to San Antonio, for the benefit of his health, upon advise of Drs. Loomis, Da Costa and others. Dr. Bell was born and raised in Austin, we believe, and is a son of the late Judge Bell of this city. We learn that the doctor will engage in the practice of his specialty, and the JOURNAL extends to him a cordial welcome to Texas and the best wishes for his health and prosperity.

---

**The Climate of Texas and Surgical Operations.**—Dr. E. J. Beall, of Fort Worth, or rather Drs. Beall, Walker & Capps, prepared a paper, which was read before the last meeting of the Texas State Medical Association, entitled "The Fitness of the Climate of Texas or Operative Surgery, Demonstrated by Results in Recent Capital Cases." It is a most interesting paper, and in it the author not only "demonstrates the fitness," but the *advantages* the climate of Texas offers, and fully refutes the assertion to the contrary, industriously circulated by persons interested in having patients leave Texas and go to — elsewhere to be operated on. We not only have the *climate*, but we have the *surgeons.*

---

**"As Others See Us."**—Says an old time subscriber: "I have just compared Vol. X. No. 1, with Vol. I. No. 1 of the now immortal 'Red Back,' and it is—well—strictly '*outer site.*' Your success has indeed been 'phenomenal,' but the best of all is, you have *fulfilled your promises, all of them*, when you introduced yourself with a bow to the medical profession of this great State and 'launched' the 'frail bark.' I could, and honestly and truthfully, say much more, but you might think I am giving you 'taffy.' So long—best wishes."

Judge Clark, Proctor Texas University, a highly esteemed patron and great admirer of the Red Back, says:

"THE JOURNAL not only has matter in it, but it is ALIVE, ALERT, VIGOROUS; traits possessed by very few medical publications. During all last session it was read by many students, and they would frequently ask me if it had come. With best wishes, I am yours as long as I last. J. B. C."

